# Safer Online Lives: Internet Use and Online Experiences of Adults With Intellectual Disabilities—A Survey Study

**DOI:** 10.1111/jar.70061

**Published:** 2025-04-30

**Authors:** Paraskevi Triantafyllopoulou, Jessie Newsome, Winnie Tsang, Michelle McCarthy, Karen Jones

**Affiliations:** ^1^ Tizard Centre University of Kent Canterbury UK; ^2^ Personal Social Services Research Unit University of Kent Canterbury UK

**Keywords:** disability, intellectual disability, internet, learning disability, online, social media

## Abstract

**Background and Aims:**

The internet and social media are increasingly accessible to people with intellectual disabilities, offering significant benefits but also posing unique challenges and risks. This study aimed to explore the online experiences of adults with intellectual disabilities in England.

**Methods:**

An accessible survey was conducted from July 2021 to July 2022 collecting data on online risks, perceived benefits and barriers to internet use.

**Results:**

Amongst 115 participants, 74% reported using the internet daily, and 48% used social media every day. Participants who reported more frequent engagement in cyber‐aggression were significantly more likely to also report experiences of cyber‐victimisation.

**Conclusions:**

The findings highlight the evolving use of the internet for adults with intellectual disabilities and the factors that influence their online experiences. They emphasise the need for strategies to enhance safe internet use and inform social care practices aimed at fostering positive online experiences whilst mitigating risks.

**Summary**

- Many people with intellectual disabilities use the internet and social media every day. However, difficulties such as hard‐to‐use websites, difficult wording and restrictions limiting their access make it harder for them to use the internet.
- The internet can help people with intellectual disabilities communicate, make friends, be independent and find information. But problems like cyberbullying and scams can put them at risk and make it harder for them to use the internet safely. It is important to understand the problems people with intellectual disabilities face online to help them use the internet with more independence.
- The study highlights the need for accessible, targeted training to help individuals with intellectual disabilities safely navigate the online world, urging policymakers, caregivers and social care providers to prioritise safe and inclusive digital access.

## 1 Introduction

The internet and social media have become integral parts of daily life, influencing how individuals interact, access information and healthcare and participate politically (European Commission 2024; Carretero‐Gomez et al. 2017). The necessity of internet access for social participation was further exacerbated by the restrictions imposed during the COVID‐19 pandemic (Chadwick et al. 2022; Caton et al. 2022; Zaagsma et al. 2020). In response, various local and national organisations in the UK facilitated internet and digital access for people with intellectual disabilities (de Beltran Guevara 2023; Mencap. 2022). In England, approximately 2.16% of adults are estimated to have an intellectual disability (Hatton et al. 2016), with most living at home with their parents (Emerson et al. 2012). Intellectual disabilities are characterised by difficulties in intellectual and adaptive functioning, which can affect various aspects of daily life (American Psychiatric Association 2013). Recent research indicates that adults with intellectual disabilities are using the internet for various purposes, including socialising, accessing information and finding entertainment (Danker et al. 2023; Chadwick et al. 2022).

Having a disability has been closely associated with lower rates of internet use (Helsper and Reisdorf 2017). Despite the apparent increase in internet use amongst people with intellectual disabilities, digital exclusion continues to disproportionately affect this population (De Castro et al. 2023; Engwall 2023; Glencross et al. 2021). People with intellectual disabilities are a heterogenous group (Nakken and Vlaskamp 2007) and face several barriers and inequalities when accessing the internet. Challenges include lack of accessibility (Ågren et al. 2023), lack of ownership of digital devices (Chadwick et al. 2013), the type and severity of intellectual disability (Johansson et al. 2021), low literacy levels (Caton and Chapman 2016) and gatekeeping by carers (Heitplatz et al. 2022; Chiner et al. 2017; Molin et al. 2015), all of which restrict access to the internet's potential benefits.

The internet offers a wealth of opportunities that can enrich the lives of people with intellectual disabilities, who may experience challenges such as social isolation and loneliness (Robinson and Idle 2022; Merrells et al. 2019). However, the internet also presents unique risks for vulnerable populations, including adults with intellectual disabilities, who are considered to be at greater risk of online victimisation (Chadwick 2022; Fogden et al. 2016; Kirwan and Power 2013). Conversely, previous qualitative research has also shown that adults with intellectual disabilities are aware of these online risks but require additional support in developing their digital skills (Chadwick 2022).

One of the risks that people with intellectual disabilities often encounter is cyberbullying, defined as the use of technology to intentionally harm, annoy or defame others (Kowalski and Limber 2013). Cyberbullying is carried out through electronic means such as email, social media, digital images, messaging, online gaming and other digital platforms (Kowalski et al. 2014). Previous qualitative research has shown that children and adults with intellectual disabilities often experience various forms of cyber‐victimisation—the act of being victimised through technology (Eroglu et al. 2022), including trolling, hacking and online sexual exploitation (Chadwick 2022; Sallafranque‐St‐Louis and Normand 2017; Bannon et al. 2015). Some individuals also engage in risky online behaviours such as illegal downloading (Chadwick 2022), and have been reported to be more likely to engage in risky situations that involve social interaction (Lough and Fisher 2016). However, no studies 2 to date have reported people with intellectual disabilities as cyber‐aggressors who use technology to harm or harass others. Although the online support needs of individuals with intellectual disabilities may vary depending on their circumstances and level of intellectual disability, gaining a comprehensive understanding of their online activity is essential for organising targeted training for this population.

With the recent introduction of the UK Online Safety Bill (Department for Digital, Culture, Media and Sport 2022), promoting internet use and safety has gained greater importance for adult social care providers. Supporting people with intellectual disabilities to gain access to the benefits of the online world and reduce the digital divide must be accompanied by a recognition of the unique risks they may face online. Although a few studies investigated the online self‐reported experiences of adults with intellectual disabilities before or during the COVID‐19 pandemic in England, the majority of these studies were qualitative in nature (Chadwick et al. 2023; Chadwick 2022; Hebblewhite et al. 2022; Lines et al. 2021). One survey study explored a broader range of experiences amongst people with intellectual disabilities during the pandemic, including basic information on internet use within this population (Caton et al. 2022). However, there is limited, up‐to‐date information on how adults with intellectual disabilities have been interacting with the online world since COVID‐19.

The current survey study explores the self‐reported online experiences of adults with intellectual disabilities in England following the COVID‐19 pandemic. Specifically, it examines how adults with intellectual disabilities use the internet, the opportunities they encounter, as well as the risks and potential barriers they face. Accordingly, the following research questions have been formulated:

- In what ways have adults with intellectual disabilities been using the internet following the COVID‐19 pandemic?
- What barriers, risks and opportunities do adults with intellectual disabilities encounter when using the internet?
- What are the experiences of adults with intellectual disabilities regarding cyberbullying victimisation and cyber‐aggression?

## 2 Method

### 2.1 Participants

Adults with intellectual disabilities living in England were invited to take part in the survey. Participants were required to currently use or have previous experience using, the internet. Following screening and cleaning of the data, a total of 115 responses were collected. This included 56 males, 57 females and 2 people who chose not to disclose their sex. Respondents were aged between 18 and 77 years (*M* = 37, SD = 14.5). The majority of the participants identified as white (80%), with only 6% and 5% of the participants identifying as Asian/Asian British and Black/Black British/African or Caribbean, respectively. 80% lived in the South of England, and most of the participants lived with family (37%). 30% lived on their own and 18% lived in supported accommodation or residential placement. 53% had attended mainstream or special educational needs college, and 57% had a job (36% a paid job, and 21%, a voluntary job).

### 2.2 Materials

#### 2.2.1 Survey

An easy‐read survey was developed to gather quantitative data on participants' internet use and online experiences. Participants were given the option to complete the survey either with or without pictures and licenced pictograms (a copy of the survey can be accessed in the Data S1). To ensure maximum accessibility, they were also given the choice to complete the survey online via Qualtrics, download a printable copy or have a copy posted to them. The survey comprised five blocks of questions: demographic, internet use, barriers, risks and opportunities/benefits.

##### 2.2.1.1 Demographic Questions

The first block of items included 12 demographic questions, such as age, sex, accommodation, ethnic background, employment and education.

##### 2.2.1.2 Internet Use and the Adapted Facebook Intensity Scale (FIS)

The second block of survey items concerned the participants' use of the internet. An initial seven items asked participants about what they do online, and what devices they use to access the internet. This block also included an adapted version of the FIS (Ellison et al. 2007) previously used to measure autistic adults' social media usage and attitudes (Triantafyllopoulou et al. 2022). The eight items of the adapted FIS were reduced to six items, as two of the original items (Q7 and Q8) were better represented elsewhere in the survey. Small changes were also made to the sentence structure of Q4, based on feedback from our advisory group (*I feel out of contact with people when I have not logged into social media*). Participants were presented with six self‐report individual item statements and were asked to respond to each statement using a three‐point Likert scale (1 = Disagree, 2 = I'm not sure and 3 = Agree). The FIS originally used a five‐point Likert scale; however, previous literature demonstrates that a three‐point Likert scale is more appropriate for use with people with intellectual disabilities (Fang et al. 2011). The internal consistency of the adapted FIS was acceptable, with a Chronbach's alpha of 0.84. Following the adapted FIS, participants were asked five further questions relating to their frequency of internet and social media usage. One of these questions investigated their use of the internet since COVID‐19.

##### 2.2.1.3 Barriers to Internet Use

Following this, participants were asked questions relating to the barriers they may face when accessing the internet. All items in this section were based on previous literature (Caton and Chapman 2016; Molin et al. 2015; Seale 2014). The first four questions related to whether the participants' internet use was restricted and, if so, why this was the case (e.g., ‘*Is your access to the internet restricted? Restricted means that there are some limits on how you can use the internet. For example, can you only use the internet at certain times, or to view certain things*?’). This theme arose from previous studies (Molin et al. 2015; Seale 2014) which identified that parents and carers sometimes acted as gatekeepers to the internet, controlling digital access for people with intellectual disabilities due to their perceived risk of harm. Ten items were also developed on the basis of a systematic review which highlighted the barriers preventing people with intellectual disabilities from accessing social media (Caton and Chapman 2016). These themes were adapted to represent the internet as a whole, rather than just social media (e.g., ‘*Paying for internet is too expensive’* and ‘I don't always have someone to help me with the internet when I need it’).

##### 2.2.1.4 Risks and the European Cyberbullying Intervention Project Questionnaire (ECIPQ)

The next block of questions dealt with the risks of using the internet. This block included two sections. Participants were first asked about how frequently they might have encountered certain online risky occurrences (e.g., ‘*Someone asked me to send them intimate/private pictures or videos of myself’*). The 11 items included in this section were inspired by and adapted from those used in Chadwick et al.'s (2017) study. The current study was interested in participants' actual experiences of online risky scenarios, rather than their perceived risk of such things happening, which was the objective in Chadwick et al. (2017). Therefore, participants were asked to indicate if they had experienced these risks using a 3‐point Likert scale: ‘*Yes, more than once’*, ‘*Yes, once*’ and ‘*No*’.

A modified version of the ECIPQ was then presented to participants. ECIPQ (Brighi et al. 2012) was used to assess experiences of cyberbullying victimisation (e.g., ‘*Someone said nasty things to me or called me names on the internet*’) and cyber‐aggression (e.g., ‘*I said nasty things about someone to other people online*’), with 11 items for each. The wording of the items was adapted after consultation with the study's advisory group, to ensure comprehension for people with intellectual disabilities. The ECIPQ has demonstrated adequate reliability for both cyberbullying victimisation (*α* = 0.97) and cyber‐aggression (*α* = 0.93) (Del Rey et al. 2015). Internal consistency for the ECIPQ was assessed using Cronbach's alpha. The cyberbullying victimisation subscale showed acceptable reliability (alpha = 0.774), indicating consistent measurement accross items within each domain. Although the original scale used a five‐point Likert format, participants in this study were asked to indicate how often each statement happened to them or how often they participated in each cyberbullying activity using a three‐point Likert scale (1 = *Never*, 2 = *Sometimes* and 3 = *Often*) as recommended by the literature (Fang et al. 2011). This modified scale produced a minimum score of 11 and a maximum score of 33.

##### 2.2.1.5 Benefits and Opportunities

Lastly, participants were asked about the benefits and opportunities of using the internet. The 24 items included in this section were inspired by and adapted from Chadwick et al.'s (2017) study (e.g., ‘*The internet helps me to make decisions about my life*’). The research by Chadwick et al. (2017) employed a five‐point Likert scale to indicate the degree to which participants perceived each item to be beneficial to them. Mindful of the population, the present study adopted a three‐point Likert scale (Fang et al. 2011) ranging from ‘*High benefit*’ to ‘*Low benefit’*. An option was also given for participants to indicate if the item was not relevant to them, i.e., they did not engage in the activity described—*‘I don't do this'*. Internal cosistency was assessed using Chronbach's alpha. The benefits and oportunities scale showed excellent reliability (alpha = 0.91), indicating a high level of internal consistency among the items.

### 2.3 Procedure

An advisory group consisting of people with intellectual disabilities was consulted throughout the research, ensuring the relevance and accessibility of the questions included in the survey. Additionally, the advisory group members piloted the survey to further assess its clarity and appropriateness. Promotional materials for the survey and interviews were designed and shared through a variety of mediums. NHS trusts acting as Participant Identification Centres (PICs), service providers supporting people with intellectual disabilities and Local Authorities all over England promoted the study to eligible participants by spreading the word about the research and displaying promotional materials. Study information was also shared on social media, online forums and wider networks of intellectual disability. The survey was available online via a hyperlink; however, participants were also given the option to download a printable copy or have a copy posted to them. Survey data was collected between July 2021 and July 2022. All participants were presented with an easy‐read information sheet detailing the study background and what would happen if they decided to take part. Participation was anonymous, but people were required to give their consent before they accessed the survey. A debrief sheet, including support organisations for people with intellectual disabilities, was available to all participants at the end of the survey.

### 2.4 Ethics Statement

Ethical approval was issued by the NHS Health Research Authority and Health and Care Research Wales (REC reference: 21/LO/0251). Informed consent was provided by all participants before accessing the survey.

### 2.5 Analysis

To investigate internet and social media use for adults with intellectual disabilities as well as the risks, barriers and opportunities people come across online, descriptive statistics were calculated and summarised, using SPSS 26. To examine the occurrence of cyberbullying victimisation and cyber‐aggression, descriptive statistics were again calculated and summarised. Nonparametric Kruskal–Wallis tests were performed to determine whether ECIPQ cyber‐aggression scores differ in relation to whether or how often participants used social media and whether they had also experienced online risks. Post hoc (Mann–Whitney *U*) tests were also performed to determine the direction of the results. Shearman's rank correlation analysis was used to investigate relationships between ECIPQ cyber‐aggression and cyberbullying victimisation scores, and a linear regression analysis was performed to predict the extent to which cyber‐aggression scores could explain the variance in cyberbullying victimisation scores.

## 3 Results

### 3.1 Use of the Internet and Social Media

The majority of the participants reported using the internet either every day (72.2%) or more than once per week (19.1%), and 79.1% of the participants reported using social media. From the participants that reported using the internet every day, 72.2% also reported using the internet up to, or more than, 2 h per day. Overall, 57.4% reported an increase in their internet use since COVID‐19. The majority of adults with intellectual disabilities reported accessing the internet via a smartphone (80.9%), a computer/laptop (77.4%) or a tablet (62.6%), and 74.8% do not have to share their device, with 94.8% accessing the internet mostly at home. Table 1 below shows the activities that adults with intellectual disabilities engage with when online.

**TABLE 1:** Online activities adults with intellectual disabilities participate in.

Online activity	*N* (%) (*N* total = 115)
Social media	91 (79.1%)
Online shopping	66 (57.4%)
Online banking	51 (44.3%)
Online courses/training	28 (24.3%)
Call friends or family	63 (54.8%)
Call paid carers (e.g., support worker)	24 (20.9%)
Online dating	10 (8.7%)
Look up information (hobbies)	58 (50.4%)
Look up information (health or social care)	41 (35.7%)
Play online games	45 (39.1%)
Chat with other gamers	18 (15.7%)
Listen to music	72 (62.6%)
Watch movies/TV shows	64 (55.7%)
Watch YouTube videos	68 (59.1%)
Read/watch the news	53 (46.1%)
Watch pornography	11 (9.6%)

Findings on the adapted FIS and data on social media (Tables 2 and 3) suggest that social media usage is part of people's everyday activity (65.8%) and part of their everyday routine for 58.6% of the participants. Additionally, 29.6% of these individuals also reported that they spend more than 2 h per day on social media. 27.8% reported that they access social media more than once per week, with Facebook being their preferred social media platform overall (70.4%).

**TABLE 2:** Social media platforms accessed by adults with intellectual disabilities.

Social media platforms	*N* (%) (*N* total = 112)	Mean *N* of followers
WhatsApp	76 (66.1%)	—
Skype	17 (14.8%)	—
Facebook	81 (70.4%)	374.25
X (Twitter)	40 (34.8%)	131.24
Instagram	54 (47%)	207.44
TikTok	25 (21.7%)	100.95
Snapchat	27 (23.5%)	63.72
Pinterest	7 (6.1%)	6.8

**TABLE 3:** Descriptive statistics for adapted FIS items.

Adapted FIS items	*M* (SD) (*N* = 111)	Participants in agreement: frequencies (%)
Social media is part of my everyday activity	2.49 (0.76)	73 (65.8%)
I am proud to tell people I am on social media	2.18 (0.84)	50 (45%)
Social media has become part of my daily routine	2.39 (0.79)	65 (58.6%)
I feel out of contact with people when I have not logged into social media	1.95 (0.85)	38 (34.2%)
I feel that I am part of a social media community	2.39 (0.78)	63 (56.8%)
I would be sorry if social media shut down	2.38 (0.8)	64 (57.7%)

### 3.2 Barriers Accessing the Internet

Participants reported multiple barriers when accessing the internet (Table 4). Some of the barriers identified were related to difficulties navigating the functionality of the internet. For example, 31.3% of participants reported challenges with the lack of internet accessibility. Other barriers, however, were more related to the individual's autonomy in choosing whether to access the internet at all. Notably, 28.4% of participants mentioned that their internet access was restricted in some way by others. A chi‐square test for independence indicated no significant association between internet restriction and their living circumstances [*χ* ^2^ (7, *N* = 107) = 4.63, *p* = 0.7]. Amongst those who experienced internet restrictions, 35% reported not knowing the reason behind these limitations. Some participants reported that their family members (25%) or their carers (3.6%) imposed restrictions on their internet use, and others indicated that a firewall (21.4%) or a care plan (14.3%) dictated restrictions on their use of the internet. From these individuals, some reported that they could only access the internet at certain times (33.3%), whereas other participants reported that could only access certain websites (66.7%) but not others, such as pornography (55%), social media (20%), gambling (10%) and shopping (10%) websites. A chi‐square test for independence revealed no significant association between participants' age [*χ* ^2^ (22, *N* = 103) = 21.92, *p* = 0.46] or educational background [*χ* ^2^ (11, *N* = 103) = 3.06, *p* = 0.99] and the barriers encountered by adults with intellectual disabilities when accessing the internet.

**TABLE 4:** Barriers accessing the internet for people with intellectual disabilities.

Reported barriers	*N* (%) (total *N* = 115)
I don't always have someone to help me with the internet when I need it.	32 (27.8%)
Someone who supports me doesn't know much about using the internet	9 (7.8%)
I find it harder to read on the internet without help	21 (18.3%)
I find it hard to spell what I want to say or search on the internet without help	28 (24.3%)
I find it hard to use the internet because of physical problems (e.g., use my hands to type)	8 (7%)
Websites on the internet can be confusing or unclear	36 (31.3%)
Websites and social media that I use sometimes change, and I find it hard to learn the new versions	25 (21.7%)
I find it hard to know what to say or how to express myself when talking online	22 (19.1%)
Paying for internet is too expensive	15 (13%)

### 3.3 Risks When Accessing the Internet

Adults with intellectual disabilities also reported on the risks they have encountered when using the internet (Table 5) with some of the participants reporting instances of multiple scams, phishing, impersonation and cyberflashing.

**TABLE 5:** Risks adults with intellectual disabilities encounter online.

Online risks	*N* (%) (total *N* = 104)
I bought something by mistake online	36 (34.7%)
Someone sent me images or videos that I did not want to see or that made me feel uncomfortable	39 (37.5%)
Someone who I have never met face‐to‐face asked me to meet them in person	34 (32.7%)
Someone asked me to send them intimate/private pictures or videos of myself	26 (25%)
Someone I did not know asked me to give them my bank details	36 (34.6%)
I gave my bank details to someone I don't know	15 (14.4%)
Someone asked me for my personal details, like my home address	35 (33.7%)
Someone who I met online tried to hurt me in real life	15 (14.5%)
I accidentally downloaded a virus onto my computer/tablet/phone	35 (33.6%)
I gambled online	20 (19.3%)
Because I was using the internet, I spent less time with friend and family, doing activities I normally enjoy	36 (34.7%)

Participants' experiences of cyberbullying victimisation and cyber‐aggression were measured using the ECIPQ (Table 6). 58.9% of adults with intellectual disabilities reported having been victims of cyberbullying, and 29.5% had self‐reported engaging in cyber‐aggressive behaviours. 28.4% of participants reported having experienced both being victims and aggressors. On the other hand, 40% of the participants had not experienced cyberbullying victimisation nor aggression. The median total cyber‐victimisation score was 13 (range = 22), and the median total cyber aggression score was 11 (range = 22). A Wilcoxon Sign‐Ranked test revealed that participants experienced significantly higher cyberbullying victimisation than engaging in cyber‐aggressive behaviours (*z* = −5.57, *p* < 0.001).

**TABLE 6:** Descriptive statistics for ECIPQ items, as reported by adults with intellectual disabilities.

Cyberbullying victimisation items (total *N* = 113)	Never *N* (%)	Sometimes *N* (%)	Often *N* (%)
Someone said nasty things to me or called me names on the internet	56 (58.9%)	32 (33.7%)	7 (7.4%)
Someone said nasty things about me to others on the internet	61 (64.2%)	23 (24.2%)	11 (11.6%)
Someone threatened me online	73 (76.8%)	16 (16.8%)	6 (6.3%)
Someone hacked into my account and stole personal information	67 (70.5%)	22 (23.2%)	6 (6.3%)
Someone hacked into my account and pretended to be me	67 (70.5%)	24 (25.3%)	4 (4.2%)
Someone created a fake account, pretending to be me	75 (78.9%)	15 (15.8%)	5 (5.3%)
Someone posted personal information about me online	81 (85.3%)	10 (10.5%)	4 (4.2%)
Someone posted embarrassing videos or pictures of me online	87 (91.6%)	5 (5.3%)	3 (3.2%)
Someone changed pictures or videos of me that I had posted online	87 (91.6%)	6 (6.3%)	2 (2.1%)
I was excluded or ignored by others on social media or internet chat room	73 (76.8%)	16 (16.8%)	6 (6.3%)
Someone spread rumours about me on the internet	78 (82.1%)	13 (13.7%)	4 (4.2%)
Cyber‐aggression items			
I said nasty things to someone or called them names using online messages	76 (80%)	12 (12.6%)	7 (7.4%)
I said nasty things about someone to other people online	82 (86.3%)	7 (7.4%)	6 (6.3%)
I threatened someone through online messages	86 (90.5%)	6 (6.3%)	3 (3.2%)
I hacked into someone's account and stole personal information	91 (95.8%)	2 (2.1%)	2 (2.1%)
I hacked into someone's account and pretended to be them	89 (93.7%)	5 (5.3%)	1 (1.1%)
I created a fake account, pretending to be someone else	89 (93.7%)	3 (3.2%)	3 (3.2%)
I posted personal information about someone online	88 (92.6%)	5 (5.3%)	2 (2.1%)
I posted embarrassing videos or pictures of someone online	90 (94.7%)	4 (4.2%)	1 (1.1%)
I changed pictures or videos of another person that had been posted online	89 (93.7%)	5 (5.3%)	1 (1.1%)
I excluded or ignored someone on social media	82 (86.3%)	10 (10.5%)	3 (3.2%)
I spread rumours about someone on the internet	88 (92.6%)	6 (6.3%)	1 (1.1%)

Further analysis was conducted to determine whether ECIPQ cyber‐aggression scores differ in relation to whether, or how often, participants had experienced risks online. A series of Kruskal–Wallis and post hoc Mann–Whitney *U* tests (summarised in Table 7 below) revealed that participants who had experienced certain risks, such as harm from someone they met online (*χ* ^2^ = 18.12; *p* < 0.001), and participants who engaged in risky behaviours, such as gambling (*χ* ^2^ = 28.10; *p* < 0.001), scored significantly higher in cyber‐aggression scores. In addition to these findings, adults with intellectual disabilities who reported that social media use was part of their everyday routine also scored significantly higher (mean rank = 52.97, median = 13.5) on ECIPQ cyberbullying victimisation when compared to peers that did not (mean rank = 31.91, median = 11) use social media every day [*χ* ^2^ (df = 2, *N* = 95, *η* ^2^ = 0.06) = 8.26], *p* < 0.05. Finally, Spearman's rank correlation revealed a strong positive relationship between ECIPQ total cyber‐aggression and cyberbullying victimisation total scores *r*(1) = 0.75, *p* < 0.001. A linear regression revealed a significant relationship between the two variables (*F* ([1], [93]) = [129.96], *p < 0.001*) with *R* ^2^ = 0.75, indicating that cyber‐aggression explained approximately 75% of the variance in cyberbullying victimisation.

**TABLE 7:** Kruskal–Wallis and post hoc analysis in relation to ECIPQ cyber‐aggression and online risks for people with intellectual disabilities.

Risk statements	Kruskal–Wallis test	Mann–Whitney *U* significance level [mean rank (median)]
Being sent unsolicited content	*χ* ^2^ (df = 2, *N* = 95, *η* ^2^ = 0.11) = 16.84***	No [41.9 (11)]*** More than once [65.88 (12)]
Request to meet strangers online	*χ* ^2^ (df = 2, *N* = 95, *η* ^2^ = 0.11) = 18.73***	No [42.11 (11)]*** More than once [68.37 (13)]
Request to send intimate content	*χ* ^2^ (df = 2, *N* = 95, *η* ^2^ = 0.11) = 24.93***	No [41.95 (11)]** Once [64.42 (13)]
		No [41.95 (11)]*** More than once [73.39 (14)]
Request to send bank details	*χ* ^2^ (df = 2, *N* = 95, *η* ^2^ = 0.11) = 17.01***	No [41.35 (11)]** Once [64.19 (12)]
		No [41.35 (11)]** More than once [58.08 (11)]
Request to send personal details	*χ* ^2^ (df = 2, *N* = 95, *η* ^2^ = 0.03) = 10.79**	No [43.1 (11)]** More than once [61.78 (12.5)]
Experienced harm in real life from someone they met online	*χ* ^2^ (df = 2, *N* = 95, *η* ^2^ = 0.13) = 18.12***	No [44.49 (11)]*** Once [77.38 (15)]
Downloaded a virus	*χ* ^2^ (df = 2, *N* = 95, *η* ^2^ = 0.08) = 8.99*	No [11.87 (3.23)]* Once [12.29 (1.82)]
		No [11.87 (3.23)]* More than once [14.91 (4.85)]
Online gambling	*χ* ^2^ (df = 2, *N* = 95, *η* ^2^ = 0.17) = 28.10***	No [11.79 (2.96)]*** Once [16.86 (3.29)]
		No [11.79 (2.96)]* More than once [13.2 (3.61)]
		Once [16.86 (3.29)]** More than once [13.2 (3.61)]
Experience isolation from friends and family	*χ* ^2^ (df = 2, *N* = 95, *η* ^2^ = 0.06) = 13.42***	No [11.89 (3.29)]*** More than once [13.9 (3.91)]
Engaging daily with social media	*χ* ^2^ (df = 2, *N* = 95, *η* ^2^ = 0.02) = 7.32*	Disagree [11 (0)]* Agree [12.63 (3.78)]

*** *p* < 0.001.

** *p* < 0.01.

* *p* < 0.05.

### 3.4 Benefits and Opportunities of Internet Use

Participants also reported on the benefits and opportunities they encounter when using the internet (see Table 8), with the majority of the participants (80%) reporting that the internet helps them keep in touch with their friends and family. Participants also reported that the internet helps them develop their IT skills (78%), helps with their communication (61%) and also keeps them informed during the COVID‐19 pandemic (70%). Sixteen percent of the participants reported that the internet helped them develop a romantic relationship, with 79% stating that this is not something that they have tried when using the internet.

**TABLE 8:** Descriptive statistics for items looking at benefits and opportunities adults with intellectual disabilities encounter when using the internet.

Benefits and opportunities statements (total *N* = 95)	I do not do this	Low benefit [*N* (%)]	Medium benefit [*N* (%)]	High benefit [*N* (%)]
Helps me keep in touch with my friends and family	9 (9.5%)	10 (10.5%)	27 (28.4%)	49 (51.6%)
I was able to make more friends	24 (25.3%)	20 (21.1%)	24 (25.3%)	27 (28.4%)
Helps me get better using the PC	7 (7.4%)	15 (15.8%)	24 (25.3%)	49 (51.6%)
I was able to talk to someone online about something I would feel awkward talking about face to face	43 (45.3%)	9 (9.5%)	21 (22.1%)	22 (23.2%)
I was able to help friends	30 (31.6%)	16 (16.8%)	25 (26.3%)	24 (25.3%)
I was able to find out about other people's opinions	21 (22.1%)	26 (27.4%)	25 (26.3%)	23 (24.2%)
I was able to get better at communicating by using the internet	12 (12.6%)	25 (26.3%)	20 (21.1%)	38 (40%)
I learned about other cultures and people	25 (26.3%)	13 (13.7%)	23 (24.2%)	34 (35.8%)
Helps me to make decisions about my life	29 (30.5%)	25 (26.3%)	23 (24.2%)	18 (18.9%)
Helps me to understand myself better	25 (26.3%)	27 (28.4%)	18 (18.9%)	25 (26.3%)
I was able to ask other people for help with my work or hobbies	31 (32.6%)	16 (16.8%)	21 (22.1%)	27 (28.4%)
I was able to feel closer to my friends	16 (16.8%)	22 (23.2%)	22 (23.2%)	35 (36.8%)
Helped me to get better at reading and writing	15 (15.8%)	31 (32.6%)	16 (16.8%)	33 (34.7%)
I was able to find about work opportunities through the internet	30 (31.6%)	17 (17.9%)	21 (22.1%)	27 (28.4%)
I was able to find out more about education or courses that I can do	27 (28.4%)	20 (21.1%)	27 (28.4%)	21 (22.1%)
I was able to be part of an online support group	33 (34.7%)	18 (18.9%)	20 (21.1%)	24 (25.3%)
I have connected with online advocacy groups	54 (56.8%)	15 (15.8%)	9 (9.5%)	17 (17.9%)
I was able to share my news with my friend through the internet	26 (27.4%)	12 (12.6%)	27 (28.4%)	30 (31.6%)
Helped me to get involved in a new hobby	25 (26.3%)	19 (20%)	24 (25.3%)	27 (28.4%)
Helped me find out information about my rights	27 (28.4%)	20 (21.1%)	19 (20%)	29 (30.5%)
I was able to use the internet to arrange to see a friend in real‐life	29 (30.5%)	14 (14.7%)	23 (24.2%)	29 (30.5%)
I found out information on COVID‐19 through the internet	16 (16.8%)	12 (12.6%)	25 (26.3%)	42 (44.2%)
I met my boyfriend/girlfriend through the internet	75 (78.9%)	5 (5.3%)	6 (6.3%)	9 (9.5%)
I was able to share my opinions on important things through the internet	40 (42.1%)	12 (12.6%)	22 (23.2%)	21 (22.1%)

## 4 Discussion

To the authors knowledge, this is the first survey study specifically developed to investigate internet use and safety for adults with intellectual disabilities and to report findings on the self‐reported online experiences of this population after the COVID‐19 pandemic. Although 72% of adults with intellectual disabilities reported accessing the internet daily, this rate is significantly lower than the national average, with 95.3% of the UK population using the internet daily in 2022 (Statista 2023a). In their COVID‐19 survey, Caton et al. (2022) also reported a higher proportion (88.9%) of people with intellectual disabilities accessing the internet daily. However, the decrease in internet use observed in the current study aligns with findings from Global Digital reports which show that global daily internet use has reverted to pre‐pandemic levels (Meltwater 2024; TechNode 2023). Unfortunately, comparable data on internet use amongst people with intellectual disabilities before the COVID‐19 pandemic is not available.

The current study's participants engaged in various activities online, with social media use being the most common activity for the majority (79.1%) and Facebook being their preferred platform (70.4%). These results align with the increase in social media use observed in the general population, post COVID‐19 (Statista 2023b). Most participants also reported feeling part of a social media community (56.8%) and expressed that they would be sorry if social media were to shut down (57.7%). Previous research has similarly highlighted positive outcomes of social media use (Anderson et al. 2023) with Chadwick and Fullwood (2018) reporting that their participants experienced a sense of autonomy and self‐worth through social media engagement.

Adults with intellectual disabilities reported various barriers to accessing the internet, with the majority citing the lack of accessibility of websites (31.3%) and literacy difficulties (24.3%), hindering their ability to engage with the online world. These findings align with previous research identifying the inaccessibility of websites (Shpigelman and Gill 2014; Williams and Hanson‐Baldauf 2010) and literacy challenges (Caton and Chapman 2016) as major obstacles for this population. Additional barriers reported by participants in the current study included internet access restrictions; 35% of participants were unsure who imposed these restrictions, whilst 25% reported that they were primarily imposed by family members. Previous studies have similarly documented instances of carers/family members implementing restrictions, gatekeeping and monitoring internet use for individuals with intellectual disabilities (Chadwick 2022; Heitplatz et al. 2021; Barlott et al. 2019; Bannon et al. 2015; Löfgren‐Mårtenson et al. 2015). Given the age of the current study's participants, such restrictions can negatively impact their sense of dignity and self‐worth, directly infringing on their human rights. In contrast, fostering positive risk taking and shared decision making should be central to supporting individuals in making informed choices. This may lead to positive outcomes, such as increased personal autonomy, independence and dignity (Seale 2014; Duerager and Livingstone 2012).

This is the first survey study to report on the online risks encountered by adults with intellectual disabilities in England. Whilst participants indicated experiencing some risky situations, such as receiving unsolicited material (34%) and instances of cyberbullying, they also acknowledged engaging in risky behaviours themselves, with 19% admitting to gambling and 14.4% sharing their bank details with a stranger, which may have resulted from grooming or a scam. Existing research supports the view that people with intellectual disabilities are often recipients of undesirable behaviour online (Chadwick 2019), including negative experiences such as scams (Darragh et al. 2017) and being the recipient of unsolicited sexual content (Jenaro et al. 2018). Although past research has noted associated risks with pursuing romantic relationships online (Chadwick et al. 2023; Normand and Sallafranque‐St‐Louis 2016), this was less evident in the current study; only 16% of the participants reported developing a romantic relationship online, whilst 65% indicated they had not used the internet for this purpose. Additionally, only 9.6% of participants reported accessing pornographic websites, though 55% noted restricted access to such content.

Instances of cyber‐aggressive behaviour involving people with intellectual disabilities as perpetrators are seldom reported in the literature. This may be attributed to the predominance of qualitative studies in this area, where participants might feel embarrassed to disclose antisocial or aggressive online behaviours to interviewers, due to a desire to maintain social desirability (Chadwick et al. 2023; Gómez‐Puerta and Chiner 2022). The current study revealed that 29.5% of participants displayed cyber‐aggressive behaviours in the past; however, their experiences of cyberbullying victimisation were significantly higher than their engagement in cyber‐aggressive behaviours. Additionally, the current study showed that participants who had encountered certain online risks, such as harm from someone they met online, or who engaged in risky activities, like gambling, scored significantly higher in cyber‐aggression. Previous studies in adolescents without disabilities suggest that victims of abuse may adopt hostility and aggression as coping mechanisms (Cook et al. 2017; Gamache et al. 2016).

It is important to highlight the benefits and opportunities available to people with intellectual disabilities through internet use. Previous studies indicate that being online can foster a sense of inclusion and enable individuals to feel ‘like everyone else’ (Raspa et al. 2018; Molin et al. 2015; Shpigelman and Gill 2014). The internet offers individuals autonomy (Kim and Qian 2021), facilitates the development of social (Danker et al. 2023) and romantic relationships (Santinele Martino and Kinitz 2022) and enables connections with a broader network beyond pre‐existing friends and family (Shpigelman 2018). Similarly, participants in the current study reported that the internet enhanced their communication, improved their literacy and IT skills, supported romantic connections and served as a valuable resource for making informed health decisions during the COVID‐19 pandemic.

Given that people with intellectual disabilities are known to face social exclusion and have limited social and support networks (Araten‐Bergman and Bigby 2022; Wilson et al. 2017), the internet has the potential to increase their social bridging capital (Shpigelman 2018). Through online interactions, they can gain financial, social and emotional resources, potentially reducing some inequalities they experience compared to their non‐disabled peers (Mithen et al. 2015; Chadwick et al. 2013).

### 4.1 Limitations

Although this is the first survey study focusing on internet use, online risks, barriers and opportunities for adults with intellectual disabilities in England, certain limitations should be noted. Participation in the study was facilitated online, which may have posed a barrier for individuals without a stable internet connection or personal device. Although paper copies of the survey and a phone call option were available upon request, these alternatives may still not have been accessible enough for some participants.

Additionally, the cognitive abilities of participants were not formally assessed. However, considering that 30% of the participants lived alone and 36% were employed, it is possible that individuals with multiple and more severe intellectual disabilities may not have been captured in the data. Whilst this is one of the largest surveys conducted with people with intellectual disabilities, the sample size and the fact that 80% of participants were from the South of England limit the representativeness of the study.

Furthermore, the dissemination of the survey occurred through organisations supporting individuals with intellectual disabilities. This could have led to a sample of individuals who received positive support and faced fewer restrictions on internet access. Finally, it should be noted that all measures in the current study are self‐reported, representing the participants' perceptions and memories of events.

## 5 Conclusions and Recommendations

The current study offers a unique quantitative perspective on internet use, online risks, barriers and opportunities for people with intellectual disabilities in England, after the COVID‐19 pandemic. Social media use was high amongst participants, highlighting its importance for this population. The study also highlighted the many benefits of internet use, including improved communication skills, literacy, IT skills and the ability to form social connections. However, a significant proportion of participants reported experiencing online risks such as cyberbullying, with some also engaging in cyber‐aggressive behaviours.

Given these findings, there is a pressing need for accessible targeted training to help people with intellectual disabilities safely navigate online environments, manage risks and maximise the benefits. The current study offers a unique quantitative perspective on internet use, online risks, barriers and opportunities for people with intellectual disabilities in England, after the COVID‐19 pandemic. Social media use was high amongst participants, highlighting its importance for this population. The study also highlighted the many benefits of internet use, including improved communication skills, literacy, IT skills and the ability to form social connections. However, a significant proportion of participants reported experiencing online risks such as cyberbullying, with some also engaging in cyber‐aggressive behaviours.

In light of these challenges, the UK Online Safety Bill (Department for Digital, Culture, Media and Sport 2022) emphasises the importance of promoting internet safety and use for vulnerable populations, including people with intellectual disabilities. This legislation underscores the necessity for specific laws and guidance related to digital inclusion and protection, making it increasingly important for adult social care providers to prioritise safe internet use for this group.

To better understand internet use, online risks and the benefits the internet has to offer this population, longitudinal research is needed as well as studies involving individuals with a broader range of cognitive abilities. Additionally, further exploration of the factors influencing cyber‐aggressive behaviours in this population would be valuable. Overall, this study underscores the internet's potential as a powerful tool for social inclusion and empowerment for people with intellectual disabilities, but also highlights the need for policies and support mechanisms that can protect them from online risks and foster positive online engagement.

## Author Contributions

**Paraskevi Triantafyllopoulou:** data collection, data analysis, ethics, write‐up of the manuscript and conceptualisation of the project. **Jessie Newsome:** data collection, data analysis, ethics and review of manuscript. **Winnie Tsang:** data analysis and review of manuscript. **Michelle McCarthy:** review of manuscript and conceptualisation of the project. **Karen Jones:** review of manuscript and consult with data analysis.

## Ethics Statement

Ethical approval was issued by the NHS Health Research Authority and Health and Care Research Wales (REC reference: 21/LO/0251).

## Consent

Informed consent was provided by all participants in this study.

## Conflicts of Interest

The authors declare no conflicts of interest.

## Supporting information


**Data S1.** jar70061‐sup‐0001‐Supinfo.

## Data Availability

The data that support the findings of this study are available from the corresponding author, upon reasonable request.
