# Supplementary material for: Safer Online Lives: Internet Use and Online Experiences of Adults With Intellectual Disabilities—A Survey Study
Source: J Appl Res Intellect Disabil. 2025 Apr 30;38(3):e70061. doi: 10.1111/jar.70061 (PMC12042067; doi:10.1111/jar.70061)
Supplement: Supplementary file 1 — Data S1. jar70061‐sup‐0001‐Supinfo. [file JAR-38-e70061-s001.docx]

| **Questionnaire** | | | | | | |
| --- | --- | --- | --- | --- | --- | --- |
| **Part 1**  **Questions about you.** | | | | | | |
|  | | | | | | |
| **What age are you?** | | | | | | |
|  | | | | | | |
|  | | | | | | |
| **Do you use the internet, or have you used it in the past?** | | | | | | |
| - Yes | | | | | | |
| - No   **If you answered no, please finish the survey here. This survey is just for people who use the internet.** | | | | | | |
| **Is someone helping you to complete this form?** | | | | | | |
| - Yes | | | | | | |
| - No | | | | | | |
|  | | | | | | |
| **If Yes, who is helping you?** | | | | | | |
| - Family | | | | | | |
| - Friend | | | | | | |
| - Carer | | | | | | |
| - Other - please tell me on the line below. | | | | | | |
|  | | | | | | |
|  | | | | | | |
| **What sex are you?** | | | | | | |
| - Male | | | | | | |
| - Female | | | | | | |
| - Prefer not to say | | | | | | |
|  | | | | | | |
| **Where do you live?** | | | | | | |
| - South West | | | | | | |
| - South East (not including Kent) | | | | | | |
| - Kent | | | | | | |
| - London | | | | | | |
| - West Midlands | | | | | | |
| - East Midlands | | | | | | |
| - East Anglia | | | | | | |
| - North West | | | | | | |
| - Yorkshire | | | | | | |
| - North East | | | | | | |
| - Other – Please tell me on the line below | | | | | | |
|  | | | | | | |
|  | | | | | | |
| \| **What is your ethnicity?**   - Asian or Asian British   Includes any Asian background, for example, Bangladeshi, Chinese, Indian, Pakistani   - Black, African, Black British or Caribbean   Includes any Black background   - Mixed or multiple ethnic groups   Includes any Mixed background   - White   Includes any White background   - Another ethnic group   Includes any other ethnic group, for example, Arab   - Prefer not to say \| \| --- \| | | | | | | |
| **What is your home like? Please choose the one that sounds most like your home.** | | | | | | |
| - I live with my family, or foster family | | | | | | |
| - I live on my own | | | | | | |
| - I live with friends or flatmates | | | | | | |
| - I live in supported accommodation | | | | | | |
| - I live in a care home | | | | | | |
| - I live in a residential placement | | | | | | |
| - I live in a hospital or rehabilitation facility | | | | | | |
| - I live somewhere else – please tell me on the line below. | | | | | | |
| 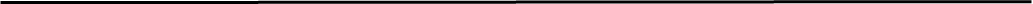 | | | | | | |
| **Have you had any education? For example, school or college.** | | | | | | |
| - Yes | | | | | | |
| - No | | | | | | |
|  | | | | | | |
| **If you answered Yes:** | | | | | | |
| **What type of education did you have? If you went to more than one, please choose the one that you went to last.** | | | | | | |
| - Mainstream school | | | | | | |
| - Special Educational Needs school | | | | | | |
| - College | | | | | | |
| - Special Educational Needs college | | | | | | |
| - Other training or education centre - Home school | | | | | | |
| - Other – please tell me on the line below | | | | | | |
|  | | | | | | |
|  | | | | | | |
| **Do you have a job?** | | | | | | |
| - Yes, I have a paid job | | | | | | |
| - Yes, I have a voluntary job | | | | | | |
| - No | | | | | | |
|  | | | | | | |
| **If you have a job:** | | | | | | |
| **Do you use the internet for your job?** | | | | | | |
| - Yes | | | | | | |
| - No | | | | | | |
|  | | | | | | |
| **Part 2**  **Questions about how you use the internet.**  You might want to take a short break from answering questions. This is okay, you can take a break if you need to. | | | | | | |
|  | | | | | | |
| **What devices do you use the internet on? You can choose as many answers as you like.** | | | | | | |
| - Computer or laptop | | | | | | |
| - Tablet or ipad | | | | | | |
| - Mobile phone or smartphone | | | | | | |
| - ipod or therapeutic device, like Brain in Hand (BIH) | | | | | | |
| - Games console | | | | | | |
| - TV | | | | | | |
| - Other – Please tell me on the line below. | | | | | | |
|  | | | | | | |
|  | | | | | | |
| **Where do you use the internet? You can choose as many as you like.** | | | | | | |
| - At school or college | | | | | | |
| - At home | | | | | | |
| - At work | | | | | | |
| - At the home of a friend or family member | | | | | | |
| - At the library or internet café - Out and about | | | | | | |
| - Other – Please tell me on the line below. | | | | | | |
|  | | | | | | |
|  | | | | | | |
| **Do you have to share the device that you use to go on the internet with other people? For example, with people that you live with.** | | | | | | |
| - Yes | | | | | | |
| - No | | | | | | |
|  | | | | | | |
| **What do you do on the internet? You can choose as many answers as you like.** | | | | | | |
| - I use social media websites, like Facebook | | | | | | |
| - I do my shopping | | | | | | |
| - I do online banking | | | | | | |
| - I do courses or training, like ASDAN | | | | | | |
| - I talk to/video call my friends or family | | | | | | |
| - I talk to/video call people who support me, like my social worker | | | | | | |
| - I go on online dating websites | | | | | | |
| - I look for information about my hobbies | | | | | | |
| - I look for information about health or social care | | | | | | |
| - I play games | | | | | | |
| - I listen to music | | | | | | |
| - I watch movies or TV shows (e.g. Netflix) | | | | | | |
| - I watch video clips (e.g. youtube) | | | | | | |
| - I read or watch the news | | | | | | |
| - I look at pornography | | | | | | |
| - Other – Please tell me on the line below. | | | | | | |
|  | | | | | | |
|  | | | | | | |
| **If you said ‘I play games’ on the internet**  **Do you chat with other gamers online when you play games?**   - Yes - No   **What game do you play the most?**  **What social media websites do you use? Please choose all that you use, and tell me how many friends/followers you have.** | | | | | | |
|  | Please tick or cross | Number of friends/followers | | |  | |
| **This is an example** | **X** | **12** | | |  | |
| Facebook |  |  | | |  | |
| Twitter |  |  | | |  | |
| Instagram |  |  | | |  | |
| TikTok |  |  | | |  | |
| Youtube |  |  | | |  | |
| Snapchat |  |  | | |  | |
| Pinterest |  |  | | |  | |
| Tumblr |  |  | | |  | |
| Flickr |  |  | | |  | |
| WhatsApp |  |  | | |  |  |
| Vsco |  |  | | |  |  |
| Skype |  |  | | |  |  |
| I do not use any |  |  | | |  |  |
| Other – Please tell me on the line below. | | | | |  | |
|  | | | | |  |  |
|  | | | | | | |
|  | | | | | | |
| **Please look at these sentences and tell me how much you agree with them.**  **Put a tick or X in just one of the boxes beside each sentence.** | | | | | | |
|  | | | | | | |
|  | | | Agree | I’m not sure | | Disagree |
| 1. Social media is part of my everyday activity. | | |  |  | |  |
| 1. I am proud to tell people I am on social media. | | |  |  | |  |
| 1. Social media has become part of my daily routine. | | |  |  | |  |
| 1. I feel out of contact with people when I have not logged into social media. | | |  |  | |  |
|  | | | Agree | I’m not sure | | Disagree |
| 1. I feel that I am part of a social media community. | | |  |  | |  |
| 1. I would be sorry if social media shut down. | | |  |  | |  |
|  | | | | | | |
| **How often do you use social media?** | | | | | | |
| - Less than once per week | | | | | | |
| - Once per week | | | | | | |
| - More than once per week, but not every day | | | | | | |
| - Every day | | | | | | |
| **If you chose ‘every day’, how many hours per day do you use social media?** | | | | | | |
| - Up to 2 hours per day | | | | | | |
| - More than 2 hours per day | | | | | | |
|  | | | | | | |

| **How often do you use the internet in general?** |
| --- |
| - Less than once per week |
| - Once per week |
| - More than once per week, but not every day |
| - Every day |
| **If you chose ‘every day’, how many hours per day do you use the internet?** |
| - Up to 2 hours per day |
| - More than 2 hours per day |
| **How much do you use the internet since Covid-19 happened?** |
| - Less than before Covid |
| - About the same amount as before Covid |
| - More than before Covid |

| **Part 3**  **These questions are about what might make it harder for you to use the internet.**  You might want to take a short break from answering questions. This is okay, you can take a break if you need to. | | | | | | | | | | |
| --- | --- | --- | --- | --- | --- | --- | --- | --- | --- | --- |
|  | | | | | | | | | | |
| **Is your access to the internet restricted? Restricted means that there are some limits on how you can use the internet. For example, can you only use the internet at certain times, or to view certain things?** | | | | | | | | | | |
| - Yes, it is restricted – there are limits to when I can use the internet, or what I can look at. | | | | | | | | | | |
| - No, it is not restricted – I can use the internet when I want to, and can look at what I want. | | | | | | | | | | |
|  | | | | | | | | | | |
| **If you said ‘Yes’**  **How is your access to the internet restricted? You can choose as many as you like.** | | | | | | | | | | |
| - I can only look at some websites, but not all of them. - I can only use the internet at certain times. - Other – please tell me on the line below. | | | | | | | | | | |
| **If you said ‘I can only look at some things, but not everything.’** | | | | | | | | | | |
| **What types of websites can you not visit? You can choose as many as you like.** | | | | | | | | | | |
| - Social media, like Facebook. | | | | | | | | | | |
| - Shopping websites, like Amazon. | | | | | | | | | | |
| - Gambling websites. | | | | | | | | | | |
| - Pornography websites. | | | | | | | | | | |
| - Film or TV websites. | | | | | | | | | | |
| - Other – please tell me on the line below. | | | | | | | | | | |
| **Why is your internet access restricted?**   - Because of my Careplan. - Because of a Firewall on the internet/it is blocked on my internet browser. - Because my carer does not like me to look at some things, even though it is not in my Careplan. - Because my family member does not want me to look at some things. - I don’t know. - Other – Please tell me on the line below.   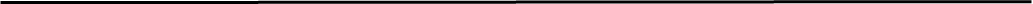 | | | | | | | | | | |
|  | | | | | | | | | | |
| **Do any of the following things ever make it harder for you to use the internet? You can choose as many as you like, or none at all.** | | | | | | | | | | |
| - I don’t always have **someone to help me** with the internet when I need it. | | | |  | | | | | | |
| - Someone who supports me **does not know much** about using the internet. | | | |  | | | | | | |
| - Someone who supports me **does not like** me to use the internet. | | | |  | | | | | | |
| - I find it **hard to read** on the internet without help. | | | |  | | | | | | |
| - I find it **hard to spell** what I want to say or search on the internet without help. | | | |  | | | | | | |
| - I find it hard to use the internet because of **physical problems**, like finding it hard to use my hands to type. | | | |  | | | | | | |
| - Websites on the internet can be **confusing or unclear.** | | | |  | | | | | | |
| - Websites and social media that I use sometimes **change**, and I find it hard to learn the new versions | | | |  | | | | | | |
| - I find it hard to know **what to say**, or how to express myself when talking online. | | | |  | | | | | | |
| - **Paying** for internet is too **expensive**. | | | |  | | | | | | |
| - None. | | | |  | | | | | | |
| - Other – please tell me on the line below. | | | |  | | | | | | |
|  | | | |  | | | | | | |
|  | | | | | | | | | | |
| **Part 4**  **These questions are about some of the risks of using the internet.**  You might want to take a short break from answering questions. This is okay, you can take a break if you need to. | | | | | | | | | | |
|  | | | | | | | | | | |
| **In the past, have any of these things happened to you on the internet?**  **Put a tick or X in just one of the boxes beside each sentence.** | | | | | | | | | | |
|  | | Yes, more than once | | | | | Yes, once | | | No |
| 1. I bought something by mistake online. | |  | | | | |  | | |  |
| 1. Someone sent me images or videos that I did not want to see or that made me feel uncomfortable. | |  | | | | |  | | |  |
| 1. Someone who I have never met face-to-face asked me to meet them in person. | |  | | | | |  | | |  |
| 1. Someone asked me to send them intimate/private pictures or videos of myself. | |  | | | | |  | | |  |
| 1. Someone I don’t know asked me to give them my bank details. | |  | | | | |  | | |  |
|  | | Yes, more than once | | | | | Yes, once | | | No |
| 1. I gave my bank details to someone I don’t know. | |  | | | | |  | | |  |
| 1. Someone asked me for my personal details, like my home address. | |  | | | | |  | | |  |
| 1. Someone who I met online tried to hurt me in real life. | |  | | | | |  | | |  |
| 1. I accidentally downloaded a virus onto my computer/tablet/ phone. | |  | | | | |  | | |  |
| 1. I gambled online. | |  | | | | |  | | |  |
| 1. Because I was using the internet, I spent less time with friends and family, doing activities I normally enjoy. | |  | | | | |  | | |  |
|  | | | | | | | | | | |
| **Please read the sentences below. For each one, please tell me how often these things happen for you.**  **Put a tick or X in just one of the boxes beside each sentence.** | | | | | | | | | | |
|  | | | | | | | | | | |
|  | | | Never | | | Sometimes | | | Often | |
| 1. Someone said nasty things **to me** or called me names on the internet. | | |  | | |  | | |  | |
| 1. Someone said nasty things **about me** to others on the internet. | | |  | | |  | | |  | |
| 1. Someone **threatened me** online. | | |  | | |  | | |  | |
| 1. Someone hacked into **my account** and stole personal information (For example through email or social media). | | |  | | |  | | |  | |
| 1. Someone hacked into **my account** and pretended to be me (For example through instant messaging or social media). | | |  | | |  | | |  | |
|  | | | Never | | | Sometimes | | | Often | |
| 1. Someone created a fake social media account, **pretending to be me** (For example on Facebook). | | |  | | |  | | |  | |
| 1. Someone posted personal information **about me** online. | | |  | | |  | | |  | |
| 1. Someone posted embarrassing videos or pictures **of me** online. | | |  | | |  | | |  | |
| 1. Someone changed pictures or videos of me that **I had posted online**. | | |  | | |  | | |  | |
| 1. **I was** excluded or ignored by others on social media or in an internet chat room. | | |  | | |  | | |  | |
| 1. Someone spread rumours **about me** on the internet. | | |  | | |  | | |  | |
| 1. **I said** nasty things to someone or called them names using online messages. | | |  | | |  | | |  | |
| 1. **I said** nasty things about someone to other people online. | | |  | | |  | | |  | |
| 1. **I threatened** someone through online messages. | | |  | | |  | | |  | |
|  | | | Never | | | Sometimes | | | Often | |
| 1. **I hacked** into someone’s account and stole personal information (e.g. through email or social networking accounts). | | |  | | |  | | |  | |
| 1. **I hacked** into someone’s account and pretended to be them (e.g. through instant messaging or social networking accounts). | | |  | | |  | | |  | |
| 1. **I created** a fake account, pretending to be someone else (e.g. on Facebook or MSN). | | |  | | |  | | |  | |
| 1. **I posted** personal information about someone online. | | |  | | |  | | |  | |
| 1. **I posted** embarrassing videos or pictures of someone online. | | |  | | |  | | |  | |
| 1. **I changed** pictures or videos of another person that had been posted online. | | |  | | |  | | |  | |
| 1. **I excluded** or ignored someone in a social networking site or internet chat room. | | |  | | |  | | |  | |
| 1. **I spread** rumours about someone on the internet. | | |  | | |  | | |  | |
|  | | | | | | | | | | |
| **Part 5**  **Questions about the opportunities and benefits of using the internet.**  You might want to take a short break from answering questions. This is okay, you can take a break if you need to. | | | | | | | | | | |
|  | | | | | | | | | | |
| **Please read these sentences. For each one, tell me how much these things benefit you. If you don’t do the thing being talked about, you can tick ‘I don’t do this’.**  **Put a tick or X in just one of the boxes beside each sentence.** | | | | | | | | | | |
|  | | | | | | | | | | |
|  | I don’t do this | | | | Low benefit | | | Medium  benefit | | High benefit |
| 1. The internet helps me keep in touch with my friends and family. |  | | | | 1 | | | 2 | | 3 |
| 1. I was able to make more friends. |  | | | | 1 | | | 2 | | 3 |
| 1. The internet helps me to get better at using the computer. |  | | | | 1 | | | 2 | | 3 |
|  | I don’t do this | | | | Low benefit | | | Medium  benefit | | High benefit |
| 1. I was able to talk to someone online about something that I would feel awkward talking about face-to-face. |  | | | | 1 | | | 2 | | 3 |
| 1. I was able to help friends through the internet. |  | | | | 1 | | | 2 | | 3 |
| 1. I was able to find out about other people’s opinions. |  | | | | 1 | | | 2 | | 3 |
| 1. I was able to get better at communicating by using the internet. |  | | | | 1 | | | 2 | | 3 |
| 1. I learned about other cultures and people through the internet. |  | | | | 1 | | | 2 | | 3 |
| 1. The internet helps me to make decisions about my life. |  | | | | 1 | | | 2 | | 3 |
| 1. The internet helps me to understand myself better. |  | | | | 1 | | | 2 | | 3 |
| 1. I was able to ask other people for help with my work or hobbies. |  | | | | 1 | | | 2 | | 3 |
| 1. I was able to feel closer to my friends. |  | | | | 1 | | | 2 | | 3 |
| 1. The internet helped me to get better at reading and writing. |  | | | | 1 | | | 2 | | 3 |
| 1. I was able to find out about new jobs through the internet. |  | | | | 1 | | | 2 | | 3 |
| 1. I was able to find out more about college or courses that I can do. |  | | | | 1 | | | 2 | | 3 |
|  | I don’t do this | | | | Low benefit | | | Medium  benefit | | High benefit |
| 1. Through the internet I was able to be part of an online support group (for example, Mencap’s online community). |  | | | | 1 | | | 2 | | 3 |
| 1. I have in the past connected with advocacy groups (for example, POWhER) through the internet.   (Advocacy groups help people to get their views across about important things by speaking for them or supporting them to do this) |  | | | | 1 | | | 2 | | 3 |
| 1. I was able to share my news with my friends through the internet. |  | | | | 1 | | | 2 | | 3 |
| 1. The internet helped me to start a new hobby. |  | | | | 1 | | | 2 | | 3 |
| 1. The internet helped me to find out information about my rights. |  | | | | 1 | | | 2 | | 3 |
|  | I don’t do this | | | | Low benefit | | | Medium  benefit | | High benefit |
| 1. I was able to use the internet, to arrange to see a friend in real-life. |  | | | | 1 | | | 2 | | 3 |
| 1. I found out information on COVID-19 through the internet. |  | | | | 1 | | | 2 | | 3 |
| 1. I met my boyfriend/ girlfriend through the internet. |  | | | | 1 | | | 2 | | 3 |
| 1. I was able to share my opinions on important things through the internet. |  | | | | 1 | | | 2 | | 3 |
|  | | | | | | | | | | |
| **End of survey** | | | | | | | | | | |
|  | | | | | | | | | | |
